# An Unusual Case of Bilateral Radix Entomolaris Treated With Lesion Sterilization and Tissue Repair in Primary First Molars

**DOI:** 10.7759/cureus.50837

**Published:** 2023-12-20

**Authors:** Himani Parakh, Nilima R Thosar, Monika Khubchandani

**Affiliations:** 1 Pediatric and Preventive Dentistry, Sharard Pawar Dental College and Hospital, Datta Meghe Institute of Higher Education and Research, Wardha, IND

**Keywords:** primary molars, supernumerary roots, radix entomlaris, lstr, lesion sterilization and tissue repair

## Abstract

Morphological dental anomalies such as supernumerary roots are rare in primary teeth. Their location, frequency, and associated peculiar root canal morphology should be known to a pediatric dentist. It is crucial to carefully examine intraoral radiographs to ensure that such variations do not go undetected. Such variances must be identified to ensure complete cleaning and filling of all root canals. Negligence in treating all canals can result in recurrent infection or treatment failure. Understanding the anatomical variation of the tooth like radix entomolaris (RE), is important for successful dental treatment. This report presents a rare case of bilateral RE of primary mandibular first molars in middle childhood treated with lesion sterilization and tissue repair.

## Introduction

Amongst all the primary teeth, molars are the largest and they provide significant function in mastication. The number of roots in primary and permanent molars is the same [1]. Developmental dental anomalies are distinct variations from the typical tooth's color, shape, size, number, or location. Apart from the aesthetic considerations, these anomalies sometimes are the cause of dental problems and they can pose certain difficulties during dental treatment too [2]. Mandibular first molars usually have two roots and three to four canals. Due to developmental disturbances, the number of root canals and the number of roots may vary. Tratman [3] reported in 1938 that three-rooted mandibular molars are frequently seen in permanent molars but have a rare frequency of <1% in the deciduous dentition. Among them primary second molars present a higher prevalence (27.8%) than primary first molars (9.7%) [4]. Radix entomolaris (RE) is also known as an “extra third root” or “distolingual root” or “extra distolingual root.” Based on the location of its cervical portion, Carlsen and Alexandersen (1990) classified RE [5,6] as type A: the RE is located lingually to the distal root complex with two cone-shaped macrostructures. Type B: the RE is located lingually to the distal root complex with one cone-shaped macrostructure. Type C: the RE is located lingually to the mesial root complex. Type AC: the RE is located centrally between the mesial and distal root complexes.

Lesion sterilization tissue repair (LSTR) is a simple endodontic procedure that involves no or minimal instrumentation that uses a triple antibiotic mix for disinfecting the root canal [7]. LSTR aims to treat infected teeth by removing infected pulp and sterilizing the root canal area to destroy bacteria that may cause infection. Hoshino et al. (1990) [8] used metronidazole 500mg, minocycline 100 mg, and ciprofloxacin 200 mg combinations in a ratio of 1:1:1 [9]. Takushige et al. (1998) used these antibiotics in a ratio of 1:3:3. LSTR's essential principle is "do not remove or touch, and leave it." Caries, pulpitis, and root canal bacteria are all treated plus medicated by it. The idea behind LSTR is that the host's natural defense mechanisms are able to repair the damage. Bioburden can be reduced by using medications to sterilize the root canals and pulp chamber. Medication-induced sterilization will result in 20-40% cleaning activity and debridement. Three-mix macrogol and propylene glycol paste is the most frequently used by combining three antibiotics with macrogol or propylene glycol as the vehicle. It is possible to expect tissue repair if the procedure is effective [9].

## Case presentation

A nine-year-old patient in his mixed dentition phase reported decayed teeth in the lower back right and left tooth region of the jaw. On clinical examination deep proximal carious lesions were found in the right and left primary first mandibular molar. The teeth were slightly tender on percussion and grade I mobile as well. A preoperative radiograph was taken. In both the teeth the carious lesion was extending as root caries and a radiolucency was noted in the inter-radicular area. Internal resorption was also evident. Additionally, it was found that there was a developmental morphological variant having a supernumerary third root present bilaterally in these deciduous first molars. Even though the appearance of an extra cusp or bulge of RE might hint at anatomical variation, it cannot be diagnosed merely by clinically examining the crown [10]. The clinical crowns in this case are anatomically analogous to the normal anatomy of a deciduous mandibular first molar. A radiographic diagnosis plays an important role, especially in such instances.

Diagnostic procedure

**Figure 1:**
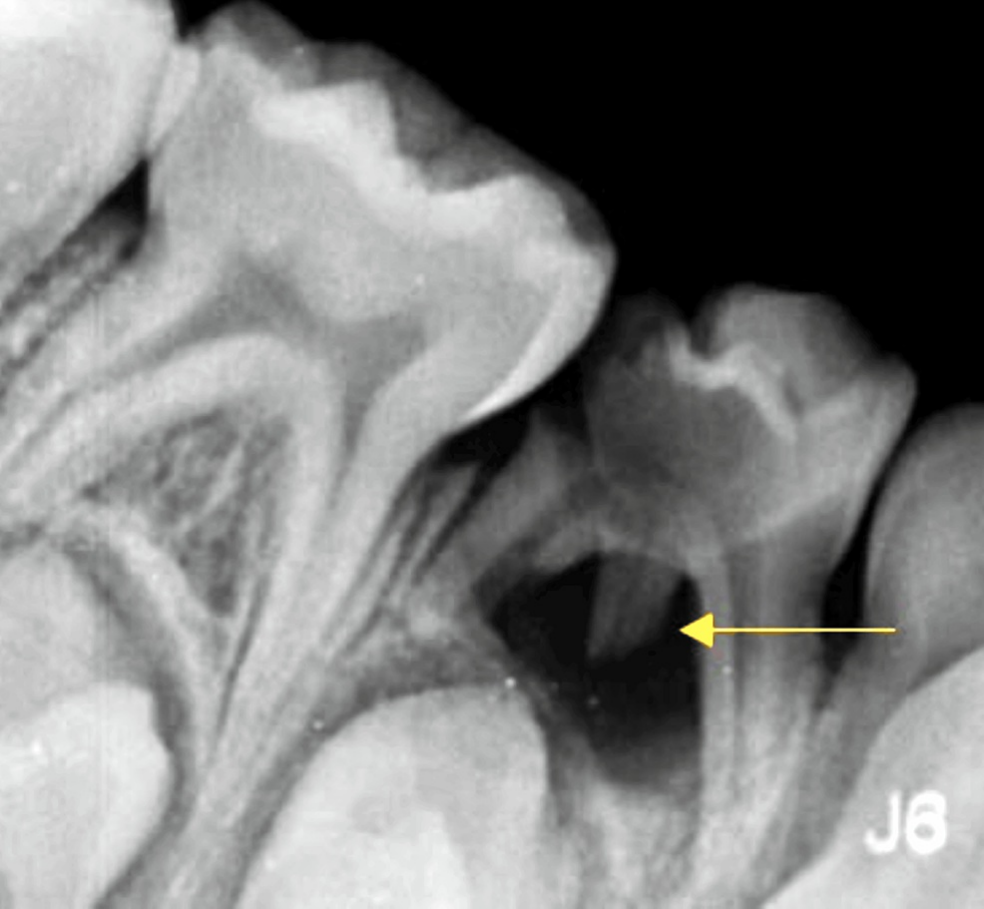
Depiction of the preoperative radiograph of RE of the left deciduous mandibular first molar RE: Radix entomolaris

**Figure 2:**
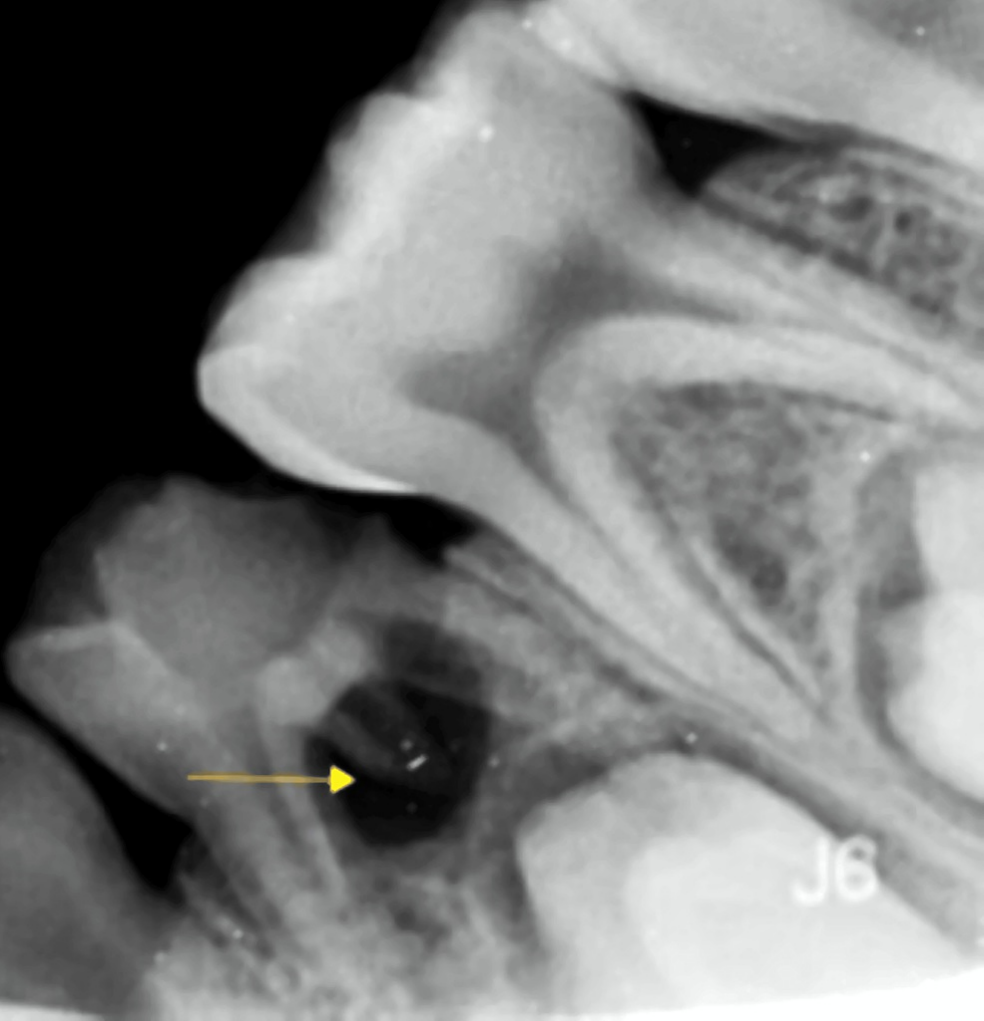
Depiction of the preoperative radiograph of RE of the right deciduous mandibular first molar RE: Radix entomolaris

The diagnosis was reached after recording a pre-operative intra-oral periapical radiograph of the lower jaw's lower right and the left molar region was recorded at normal and 30° angulation to confirm the presence of a third root (Figures 1, 2). Hence, it is fundamental to read the diagnostic radiograph carefully. In this case, RE was identified as the “AC” type showing: a central location, between the mesial and distal root complexes.

Treatment

**Figure 3:**
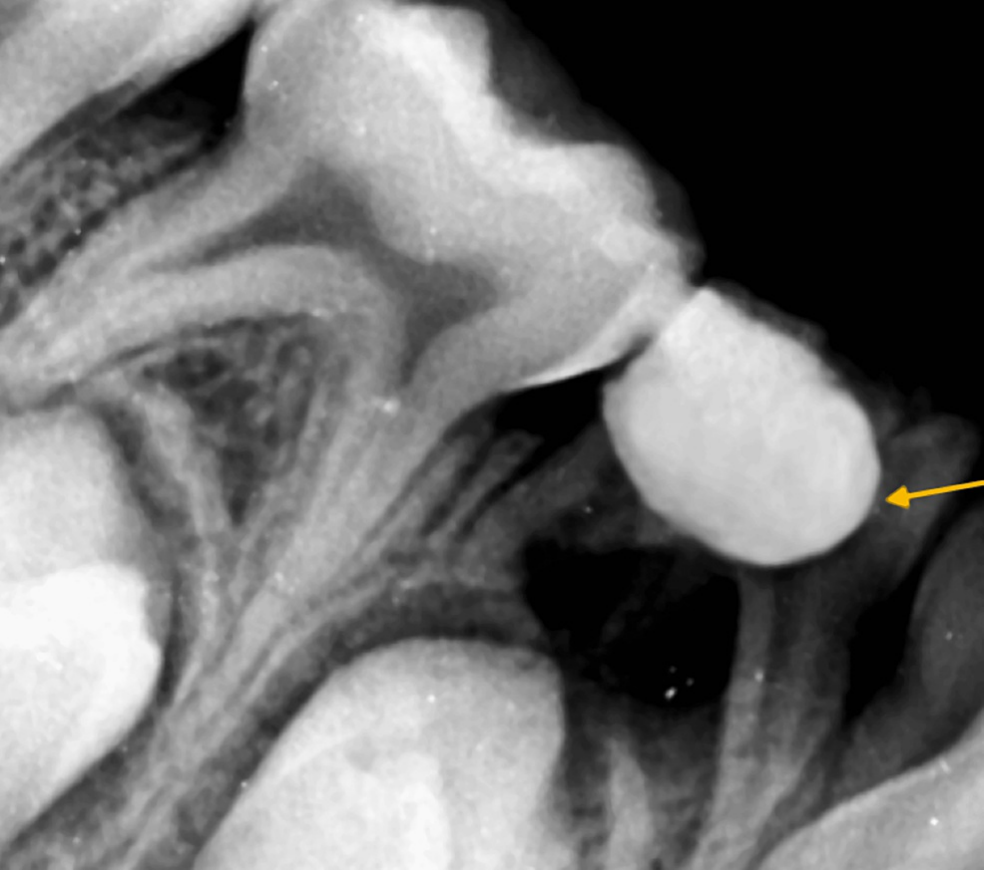
Depiction of the immediate post-operative radiograph of RE of the left deciduous mandibular first molar RE: Radix entomolaris

**Figure 4:**
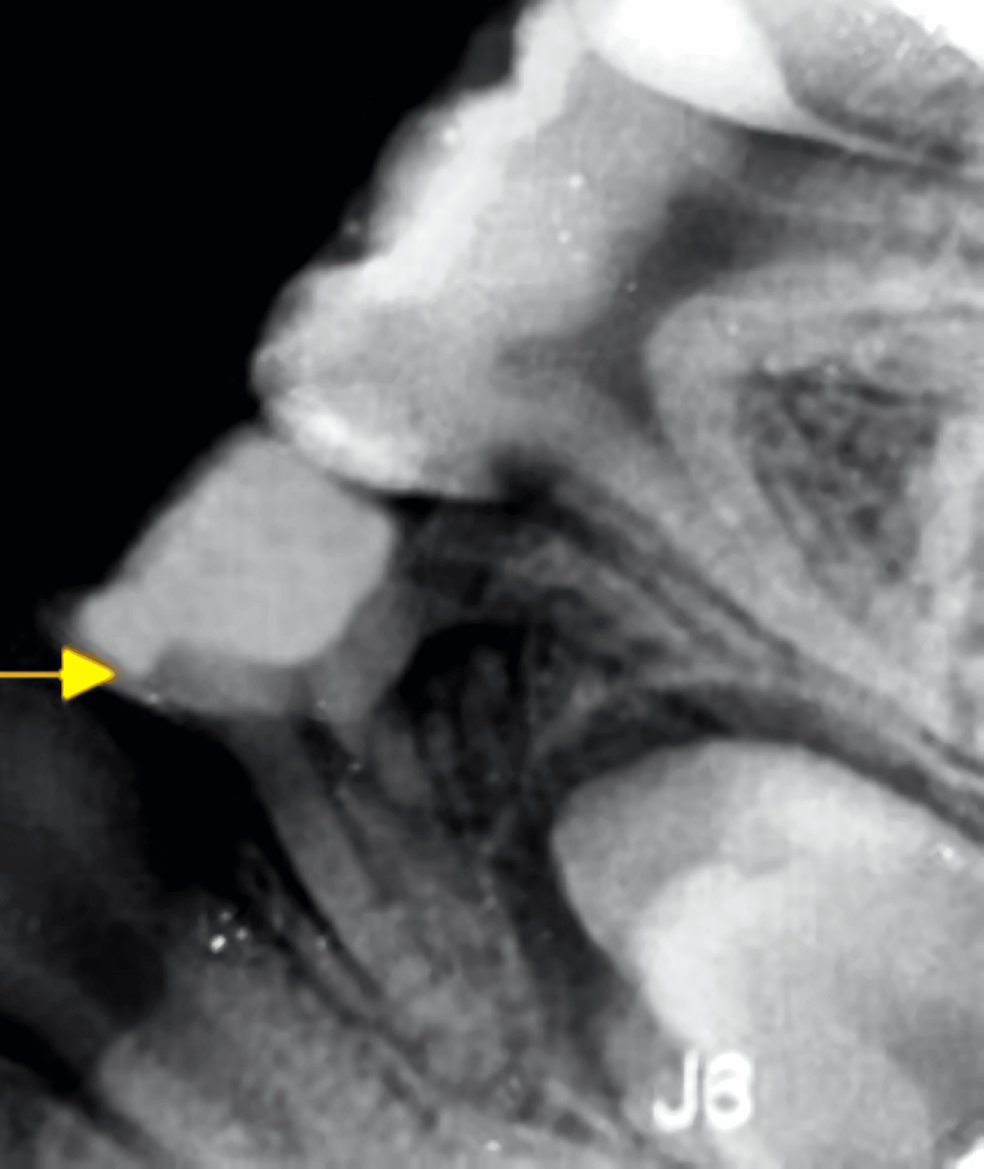
Depiction of the immediate post-operative radiograph of RE of the left deciduous mandibular first molar RE: Radix entomolaris

LSTR procedure was scheduled to be done in two appointments, beginning with the left and then the right deciduous mandibular first molar. After administration of local anesthesia, all the carious part was removed and an access cavity was made by removing the coronal pulp entirely. Thorough irrigation using sodium hypochlorite and saline was done. A medication cavity of 1 mm diameter and 2 mm depth was prepared at the orifices of the canal using a round bur to receive the triple antibiotic paste (TAP). Ciprofloxacin (200 mg), metronidazole (400 mg), and minocycline (100 mg) that is available commercially were used in a 1:3:3 ratio. This combination was admixed with propylene glycol to obtain a paste-like consistency in which one part of the vehicle was used to mix approximately five parts of the powder. Sealing the dentine tubules with a bonding agent was done to avoid tooth discoloration by minocycline. Then the TAP was then placed in the medication cavity. A base of zinc oxide eugenol paste was placed over it. Final restoration with type IX glass ionomer cement was done (Figures 3, 4). The same steps were followed for both right and left radix molars.

**Figure 5:**
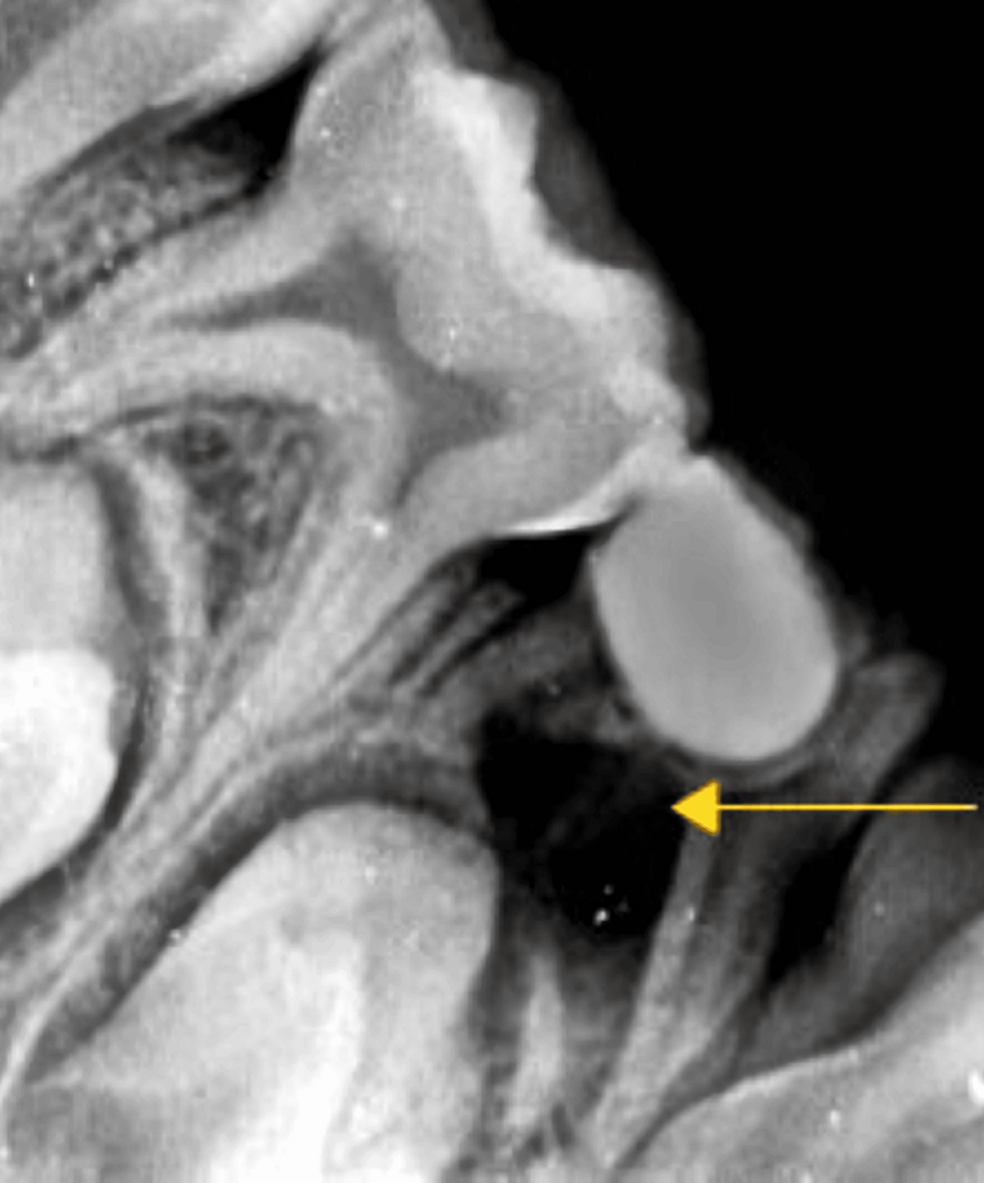
Depiction of the radiograph of RE of the left deciduous mandibular first molar at nine-month follow-up RE: Radix entomolaris

**Figure 6:**
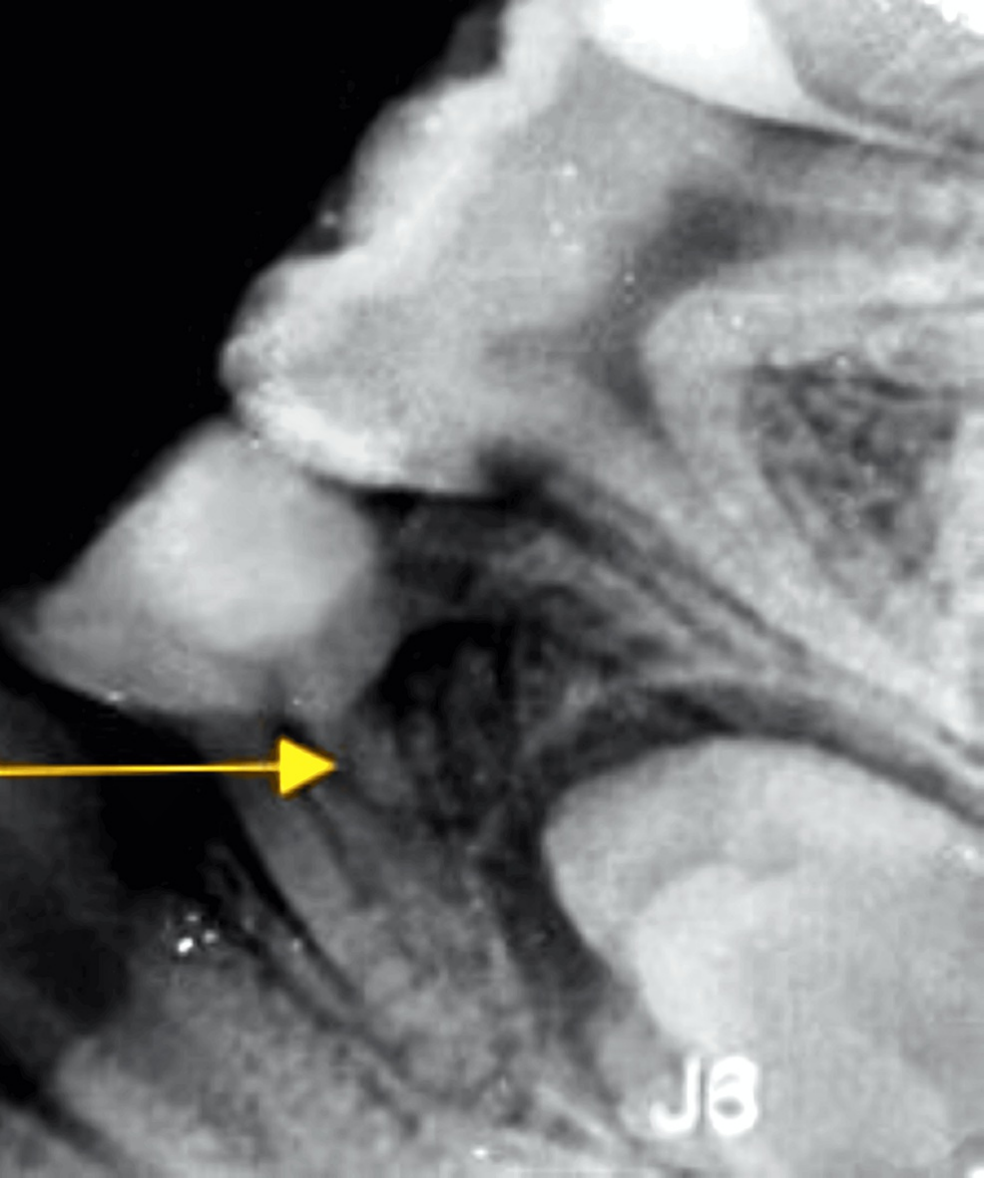
Depiction of the radiograph of RE of right deciduous mandibular first molar at nine-month follow-up RE: Radix entomolaris

The follow-up was taken at 1 month, 3 months, 6 months, and 9 months. The patient was symptom-free at all the follow-up periods. As an outcome of the treatment employed with LSTR, clinically no sign of intra-oral abscess, swelling, or pain was noted, the grade 1 mobility also disappeared and the radiograph revealed a reduction in the inter-radicular radiolucency by six months. The success rates showed a progressive improvement within nine months (Figures 5, 6). Hence the tooth was restored to normal function.

## Discussion

Poor assessment and management of supernumerary roots in molars may result in endodontic treatment failure which may lead to early tooth loss, creating altered functionality. Usually, the distal root and the RE lie in the same buccolingual plane. Therefore on a diagnostic radiograph, superimposing roots might be seen, resulting in an incorrect interpretation. To confirm the supernumerary root, another radiograph with a greater mesial or distal horizontal angulation (20-30 degrees) proves useful. After thorough evaluation conventionally a pulp therapy procedure is carried out keeping in mind the anatomical variation followed by a stainless steel crown. The best option for saving a primary tooth with pulpal involvement along with symptoms is pulpectomy. In this case, the prognosis of the treatment was poor as there was grade one mobility seen clinically, and radiographically radiolucency was evident in the inter-radicular area, also internal resorption was seen with the left primary mandibular first molar hence achieving a cleaned and shaped canal for obturation was difficult. The parents were informed about the treatment of exodontia, because of the poor prognosis with pulpectomy. The parents were not willing to extract the teeth, and taking into consideration the age of the patient as another factor the teeth were not to be retained for more than 12 months ahead hence it was decided to proceed with LSTR. According to the systematic review and meta-analysis by Coll et al., success rates for pulpectomy in the teeth having neither external nor internal root resorption were 92% and that for LSTR was 65%. LSTR is superior to pulpectomy in teeth with root resorption or when the teeth that would undergo extraction have to be preserved for up to one year [11]. LSTR is a useful technique in treating infected teeth, but the decision to use this technique is based on various factors, including the extent of the infection, patient factors, and clinician preference. In this case, the radix molars presented with intra-radicular radiolucency and internal root resorption was evident too.

Since the root canal infection contains both aerobic and anaerobic flora, disinfecting the canal completely requires the use of a mix of antibiotics rather than just one medication. Jaya et al. studied the effectiveness of metronidazole, minocycline and ciprofloxacin with minocycline, ciprofloxacin, and tinidazole clinically and radiographically with a follow-up of 24 months, it was stated that the inter-radicular lesions in primary teeth can be treated using the mixture of the latter [12].

Both aerobic and anaerobic bacteria make up the root canal's microbial flora, the anaerobic bacteria being predominant. TAP contains both bactericidal components (ciprofloxacin, metronidazole) and bacteriostatic components (minocycline) to effectively eradicate both types of bacteria [7]. Because of this action of TAP in the present case, a microbial-free environment may be formed to render sterilization of the canals despite the supernumerary root to achieve a successful treatment.

Chakraborty et al. reported a case series where LSTR therapy by TAP was done in primary molars with internal resorption and periradicular pathosis, successful results were evident at three- to six-month follow-up [13]. In this particular case instead of a conventional endodontic or exodontic procedure, after weighing all the factors that can influence the prognosis of retaining the bilateral first mandibular primary molars with RE until its timely exfoliation, a novel approach of LSTR was attempted and successful results were seen at nine-month follow up. LSTR is a non-invasive technique used mainly for primary teeth, but it can also be used for permanent teeth with non-critical pulpal exposure. This technique may be recommended for patients with poor oral hygiene, low dental intelligence quotient, or limited access to dental care. It eliminates the need for advanced equipment or trained endodontists, making it a viable option in underserved areas.

LSTR's major benefit is that it may be done in a single visit. Additionally, it is easy, painless, and quicker, and these factors are important especially while treating the pediatric population. In situations when LSTR was used, it was found that repair of the bone takes place [14]. Considering the promising outcomes of the RE with LSTR continued research is required for LSTR to become a reliable treatment option.

## Conclusions

For a better clinical approach and to avoid procedural errors, the diagnosis of developmental anomalies especially the ones with root variations is essential. Appropriate radiographic investigations are necessary for a definitive diagnosis of RE. An approach like LSTR that is less invasive and less time-consuming may serve as a silver lining to the patient and the pediatric practitioner in the 21st century. Understanding the treatment options available for such teeth will aid in retaining them successfully in the oral cavity for as long as functionally possible.
